# Report on the Recent Appearances Observed Post-Mortem in a Case of Delirium Grave

**Published:** 1883-11

**Authors:** S. V. Clevenger

**Affiliations:** Special Pathologist, Cook County Insane Asylum, Chicago, Ill.


REPORT ON THE RECENT APPEARANCES OBSERVED POST MORTEM IN A CASE OF DELIRIUM GRAVE. BY S. V. CLEVENGER, M.D., Special Pathologist, Cook County Insane Asylum, Chicago, Ill.

Mary Ryan (case book No. 275) was admitted to this hospital June 28, 1883. Her brother, Patrick McD., visited her soon after, and from him was obtained all that is known of her previous history.

The patient, æt. 22, was a happy, innocent girl, living on her father's farm, near Dublin, when in January, 1882, she was married and left with her husband for New York ; thence they came to Chicago, where her husband deserted her on the streets, leaving her with only three dollars, *enceinte*, and without a friend in the city. In April, 1883, Patrick, who was in St. Louis, received a letter from his sister stating the circumstance, and that she was quite destitute, begging for help. He immediately wrote to her, inclosing money. As he obtained no answer to several letters written to her, he came to Chicago to find her in jail, insane, just before her admission here. She had given birth to a girl baby at the county hospital seven weeks before. The distress of the brother was very great, and as bearing upon the possible heredity predisposition it is worth mentioning that he did not appear to me to be capable of enduring a great amount of suffering without risk of mental unhinging. He was a laborer, mannerly and quiet, evidently well brought up and disposed.

The patient raved incessantly. Her attention could be attracted for an instant only. She recognized her brother, but the next moment lost all interest in him. Her restlessness was so extreme as to necessitate restraint to prevent her harming herself. She articulated sentences intelligibly enough, but incoherently. There appeared to be no particular thread to her utterances any more than in terminal dementia. Insomnia was so profound that if she slept at all while here, the attendants remained unaware of it. Dr. J. C. Spray, the medical superintendent, was unremitting in his efforts to quiet and sustain her, but the usual sedatives had no effect whatever, and her condition grew rapidly worse, she developing the ordinary signs of acute delirium. Milk was fed to her freely, stimulation continuously tried, but without avail. July 9, she was too exhausted to leave her bed, and twelve hours before death there was marked collapse. July 13, while administering diluted brandy to her, she suddenly expired.

Twelve hours after death I made a post mortem examination of the contents of the head, and I may be pardoned for digressing enough to say that could some of those who derive all their knowledge of asylum management from novel reading and the sensational trash published in newspapers for political purposes, have seen with what womanly tenderness the attendants had arrayed the corpse, the vulgar notion that asylum officials and employès are less tender hearted than the rest of the world might have been foregone. Hypostatic congestion of the entire body was marked. Somewhat large pigmented macules, apparently simple ephelides, on hands and arms, not observable elsewhere. No pemphigus bullæ, such as Jessen (in *Allg. Zeitschrift für Pyschiatrie*, 1880) considers a somewhat constant accompaniment, and which Spitzka ("Insanity," p. 250) noted as absent in two out of five cases in his experience. Vessels of conjunctivæ absolutely empty. Veins of dura congested. Pacchionian bodies adhered to skull, which when lifted, tore through these bodies into the superior longitudinal sinus, from which the blackened blood escaped and coagulated quickly. There was a strong adhesion of membranes and cortex to calvarium in left orbito-frontal region, of the size of a dime. The entire length of the dura over superior longitudinal sinus was deeply stained a rusty color an inch wide. General basilar and temporal adhesions of dura to skull, but no other brain adhesion than the one mentioned. Beneath the dura, covering every inch of the brain to the depth of one-twentieth of an inch or more, was a heavy white exudate imparting a pearly luster which almost obscured the fissures in which it lay especially deep. The upper part of the brain and its meninges were greatly congested, the larger vessels standing out like whip cords, but the hyperæmia at the base of the brain was excessive. Ne departure from the normal observable in the disposition of the convolutions, but the entire organ was puffy, œdematous and yielding; the disintegrative processes were so advanced as to require much effort to preserve the tissues for future microscopic examination, which will be made by Dr. E. C. Spitzka, Dr. H. D. Schmidt (pathologist of Charity Hospital, New Orleans) and myself, and will be reported in articles to be published hereafter, as this journal goes to press too early for their completion.

The consultation which Dr. Spray, Dr. Thuemmler (the assistant physician), and the writer held over the progress and autopsy in this case resulted in justification of the treatment adopted and in the firm belief that the poor woman was doomed before reaching the asylum; tremendous doses of hypnotics would have been required to produce any effect, and it is questionable whether their exhibition would have been justifiable. Certainly the bromides would have but added to the trouble; ergot and chloral had no more effect than were they not given at all; stimulation was indicated with nourishment to overcome the exhaustion, as it progressed; but all will admit that stimulation exerted no beneficial influence over the hyperæmia, except in a possible *vis-a-tergo* anti-congestive way or diffusant. It would seem that whatever was done, cause and effect would travel in such a vicious circle that her speedy death was inevitable.

I am convinced that had her malady been even faintly recognized at the outset, before the extensive vaso-motor changes had supervened, and her mental perturbation been quieted at a very early stage of her insanity, by hypnotics, which would at that time have availed, she *might have* bridged over the period of fright and grief with a sound mind. As to whether the mania began with the puerperal state or before I was unable to learn.—*Journal of Neurology and Psychiatry*, August 1883.

